# Adapting Resources for Enhancing Alzheimer's Caregiver Health for Dementia-Capable Services

**DOI:** 10.1093/geroni/igaa057.179

**Published:** 2020-12-16

**Authors:** Mindi Spencer, Maggi Miller, Diana Jahries, James Davis, S Melinda Spencer

**Affiliations:** 1 University of South Carolina, Columbia, South Carolina, United States; 2 University Of South Carolina, Columbia, South Carolina, United States; 3 Center for Success in Aging, PRISMA Health, Greenville, South Carolina, United States

## Abstract

The overall goal of the PRISMA Health - REACH (PH-REACH) project was to reduce caregiver burden and improve caregiving skills among caregivers of community-living Alzheimer's disease and related dementia patients and, as a result, improve care and quality of life for both the patients and their caregivers. This evidence-based, person-centered program emphasizes positive aspects of caregiving and provides tools to improve stress management, caregiver self-care, and coping skills for managing problem behaviors. PH-REACH provided in-home caregiver training, support, and service referral to 126 caregivers in the Greenville area. Trained coaches delivered the program to caregivers of persons with moderate to severe dementia in its original format but later adapted it to better fit the caregivers’ needs. Analysis of pre- and post-test data showed that both the standard and adapted interventions provided benefits across multiple caregiver outcomes, including reduced caregiver burden, ability to manage disruptive behaviors of the care recipient, increased caregiver self-efficacy, reduced depression, and a slight improvement in the number of chronic health conditions. This supports and expands on previous research that has demonstrated the ability of this program to translate across different community-based and clinical settings. The tailored version of PH-REACH succeeded in assisting these caregivers, meeting them where they were in their caregiving journey, and provided measurable benefits to both their mental and physical health. Overall, this project provided evidence of the utility of the PH-REACH intervention and laid the groundwork to extend caregiver training and support to other institutions, both inside and outside the health system.

